# The Positivity Scale: Concurrent and Factorial Validity Across Late Childhood and Early Adolescence

**DOI:** 10.3389/fpsyg.2019.00831

**Published:** 2019-04-24

**Authors:** Antonio Zuffianò, Belén López-Pérez, Flavia Cirimele, Jana Kvapilová, Gian Vittorio Caprara

**Affiliations:** ^1^ChildLab, Department of Psychology, Liverpool Hope University, Liverpool, United Kingdom; ^2^Department of Psychology, Sapienza University of Rome, Rome, Italy

**Keywords:** positivity, p scale, late childhood, early adolescence, measurement invariance, developmental sensitivity

## Abstract

Despite the well-established protective functions of *positivity* (i.e., a dispositional self-evaluative tendency to view oneself, life, and future under a positive outlook) from middle adolescence to old age, its reliable assessment and contribution to a proper psychological functioning have received little attention during previous developmental phases. In this article, we aimed to evaluate the psychometric properties and construct validity of the eight-item Positivity Scale (P Scale; Caprara et al., 2012) during late childhood and early adolescence in a sample of British students (*N* = 742; 48% boys) from both primary (M*_age_* = 10.75, *SD* = 0.52) and secondary schools (M*_age_* = 13.38 years, *SD* = 0.94). First, results from confirmatory factor analysis (CFA) attested to the plausibility of the hypothesized 1-factor structure of the P Scale in a revised CFA model including the correlation between the residuals of two items similar in their wording. Next, we found evidence for strong (scalar) measurement invariance of the P Scale across late childhood and early adolescence as well as for its concurrent validity as indicated by expected relations of positivity to indicators of adjustment (i.e., prosocial behavior) and maladjustment (i.e., externalizing and internalizing problems). Overall, these findings support the concurrent and factorial validity of the P Scale as a short self-report instrument to measure children’s tendency to view their experience from a positive stance. We discuss the implications of our results for improving the wording of the items composing P Scale as well as for understanding the dispositional mechanisms conducive to psychological health and wellbeing across late childhood and early adolescence.

## Introduction

Given the alarming rates of mental health problems among youth (Kieling et al., 2011), psychologists have devoted their effort to identify those individual characteristics that may sustain people’s wellbeing. Amongst them, Caprara et al. (2012) highlighted the protective role of *positivity* (POS), which refers to a dispositional tendency to view oneself, one’s own life, and future under a positive outlook (Caprara et al., 2012). In detail, POS has been proposed as the common underlying factor for self-esteem (i.e., individuals’ global self-regard; Rosenberg, 1965), optimism (i.e., the extent to which people hold positive expectations about their future; Scheier and Carver, 1993), and life satisfaction (i.e., a general evaluation about one’s own life; Diener et al., 1985). In order to assess POS, Caprara et al. (2012) developed the Positivity Scale (P Scale), a brief self-report measure of POS. The P Scale has been used in several studies that attested to the positive relations between POS and important outcomes, such as physical health and hedonic balance (Caprara et al., 2010b), quality of relationship styles (Castellani et al., 2016), positive affect (Caprara G.V. et al., 2017), prosociality (Luengo Kanacri et al., 2017), resilience (Milioni et al., 2016), and healthy functioning in old age (Caprara M. et al., 2017). Yet, despite the considerable amount of empirical evidence concerning the key role of POS from middle adolescence (Luengo Kanacri et al., 2017) to old age (Caprara M. et al., 2017), the extent to which POS could represent a protective dispositional factor during childhood and early adolescence remains partly unexplored (see Tian et al., 2018). This is relatively surprising, since the onset of mental health problems during adulthood often occur before the age of 14 years (World Health Organization [WHO], 2013). Hence, in the present study, we aimed to address this gap – first – by testing the psychometric properties of the P Scale; (Caprara et al., 2012) in a sample of children and early adolescents from the United Kingdom, and – second – by evaluating its concurrent validity with indicators of adaptive (i.e., prosocial behavior) and maladaptive behaviors (i.e., internalizing and externalizing problems).

### POS: Conceptualization and Associations With Wellbeing

In line with previous studies pointing out to the importance of individual factors conducive to high psychological wellbeing (e.g., Beck, 1967; Seligman et al., 2005), Caprara and colleagues (e.g., Caprara G.V. et al., 2017; Caprara et al., 2018) identified POS as an enduring personality tendency that may help people approach their life under a positive outlook. Similar to other personality dimensions, the trait-like nature of POS has been empirically corroborated by genetic findings showing that a large proportion of its variability (more than 50%) was accounted for by heritability factors (see Fagnani et al., 2014). In addition, longitudinal studies also demonstrated the high mean-level and rank-order stability of POS from middle adolescence to young adulthood (see Alessandri et al., 2012a).

Although more research is needed to clarify the developmental nature of POS (e.g., its emergence during childhood) and its nomological associations, previous works contributed to differentiate POS from other variables such as personality traits (e.g., Big Five), positive affect, and domain-specific evaluative constructs. For instance, whereas personality traits can be conceptualized as behavioral tendencies, POS, being primarily linked to the self-system, is a self-evaluative disposition (Alessandri et al., 2015) concerning the way in which individuals approach their life and experience by reflecting on themselves (i.e., self-esteem), their past (i.e., life satisfaction) and their future (i.e., optimism). Empirical evidence, indeed, attested to the unique positive contribution of POS to job performance and prosocial behaviors in the organizational context when the big five traits (and positive affect) were kept under control (Alessandri et al., 2012b). Similarly, POS appeared to be different from positive affect, as it likely constitutes the dispositional base to experience happiness in life. In fact, both short-term (Alessandri et al., 2014) and long-term (Caprara G.V. et al., 2017) longitudinal studies clearly indicated the consistent predictive effect of POS on positive affect over time and not vice versa. Furthermore, concerning specific evaluative constructs such as self-esteem, positive attitudes toward the future (e.g., optimism, hope), and self-evaluations of one’s own life (e.g., life satisfaction), POS can be seen as a global evaluation tendency that subsumes these constructs. Empirical studies consistently reported the stronger associations of POS with several key outcomes (e.g., health, positive and negative affect, depression, and quality of friendship) compared to its subcomponents (see Alessandri et al., 2012a; Caprara et al., 2012).

Importantly, POS has also shown to be partly malleable since both individual characteristics (i.e., people’s self-efficacy beliefs in regulating their emotional and social life; Caprara et al., 2010a) and social factors (i.e., the presence of a positive climate at school; Luengo Kanacri et al., 2017) have been found to predict higher levels of POS (or its subcomponents) over time. Hence, despite its main trait-like nature, these findings suggest that POS might be strengthened by appropriate intervention actions to sustain people’s wellbeing. As a basic disposition to approach positively one’s own experience, indeed, POS likely provides the motivational resources to help individuals cope with the challenges of life (failures, illness, loss, etc.,) and ultimately protect their mental health (Caprara et al., 2018).

In sum, the importance of POS (and the P Scale) has been attested in many research works, as POS showed positive associations with various indicators of wellbeing such as happiness (Alessandri et al., 2012a, 2014; Caprara G.V. et al., 2017), resilience (Alessandri et al., 2012a; Milioni et al., 2016), quality of interpersonal relationships (Alessandri et al., 2012a; Caprara et al., 2010b), happiness (Lauriola and Iani, 2015), prosocial behavior (Luengo Kanacri et al., 2017), and physical health across both normative (e.g., Caprara et al., 2009; Caprara M. et al., 2017) and clinical samples (see Caprara et al., 2016). We refer the readers to Caprara et al. (2018) for a more comprehensive review of the association of POS with psychological and health outcomes.

Based on these premises, we argue about the relevance of assessing the validity of the P Scale across late childhood and early adolescence as, during these developmental phases, children and adolescents likely experience identity development shifts (Kroger, 2007) and physical changes, which jointly contribute to create a period of “storm and stress” (Hollenstein and Lougheed, 2013). In addition, many children and adolescents also face difficulties at school (e.g., peer victimization), which may have long lasting effects on their mental health (McDougall and Vaillancourt, 2015). Hence, studying the validity and reliability of the P Scale across late childhood and early adolescence can help us understand whether the putative protective role of POS could be extended to early developmental stages, thereby informing intervention actions aimed at sustaining students’ mental health.

### Measuring POS: The P Scale

Although in earlier works (e.g., Caprara et al., 2010a; Alessandri et al., 2012a) POS was modeled as a latent variable capturing the shared variance between self-esteem (Rosenberg, 1965), optimism (Scheier and Carver, 1993), and life satisfaction (Diener et al., 1985), the P Scale (Caprara et al., 2012) was later developed to specifically measure individuals’ POS. The P Scale consists of 8 items (rated on a 5-point scale) covering individuals’ *perceptions of being worthy of value* (i.e., “On the whole, I am satisfied with myself,” “I feel I have my things to be proud of,” and “I generally feel confident in myself”), individuals’ *positive expectations about the future* (i.e., “I have great faith in the future,” “I look forward to the future with hope and enthusiasm,” and “At time, the future seems unclear to me” as a reverse scored item), individuals’ *confidence in receiving support from others* (i.e., “Others are generally here for me when I need them”), and individuals’ *satisfaction with their lives* (i.e., “I am satisfied with my life”). Although the P Scale taps into specific evaluative aspects of one’s own experience, results from factor analyses indicated that these facets could be traced to a single, general self-evaluative latent construct. The unidimensionality of the P Scale, indeed, has been corroborated in several studies conducted in Brazil, China, Germany, Italy, Japan, Mexico, Poland, Serbia, Spain, and United States (see Caprara et al., 2012; Heikamp et al., 2014; Borsa et al., 2015; Macías, 2015; Tian et al., 2018). Overall, the P Scale has been found to have robust psychometric properties as indicated by its good reliability coefficients (e.g., Caprara et al., 2012), its measurement invariance across gender and countries (e.g., Caprara et al., 2012; Heikamp et al., 2014), and its expected associations with theoretically relevant constructs such as positive and negative affect, depression, and personality traits (e.g., Caprara et al., 2012; Borsa et al., 2015; Tian et al., 2018). Recent findings also attested to the convergent validity of the P Scale with implicit measures of positivity (using the implicit association test), self-perceived intelligence (Costantini et al., 2019), and dispositional flow (Riva et al., 2017).

Importantly, the factor structure of the P Scale (as well as the importance of POS for a proper psychosocial functioning) has been also confirmed across several developmental phases, from adolescence (e.g., Tian et al., 2018) to the elderly age (e.g., Heikamp et al., 2014; Caprara et al., 2018). Although an in depth analysis of the longitudinal invariance of the P scale is still needed to understand the mean-level development of POS across the life span, the aforementioned works corroborated the validity of the P scale as a psychometrically sound self-report instrument from the adolescent period onward.

### The Present Study

Despite the growing amount of studies attesting to the importance of POS as a dispositional factor against mental health issues, the extent to which POS could exert its protective role during childhood and early adolescence deserves further investigation. Although a few studies considered the effect of POS during early adolescence (e.g., Tian et al., 2018), to the best of our knowledge, there is a lack of research on POS and the measurement properties of the P Scale among children. Since previous research indicated that (a) the onset of mental health issues may occur before adolescence (World Health Organization [WHO], 2013) and (b) children and adolescents tend to differ in their level of psychological wellbeing (i.e., children have higher psychological wellbeing than adolescents; see López-Pérez and Fernández-Castilla, 2018), looking at the role of POS during childhood and early adolescence may help us understand the dispositional bases of early psychological problems as well as may inform the design of intervention programs aimed at promoting mental and behavioral health from a young age (e.g., Social and Emotional Learning Programs; Collaborative for Academic Social and Emotional Learning [CASEL], 2013). From a developmental perspective, we focused on late childhood and early adolescence, as during this transitional phase, the self-system at the core of POS becomes more coherent and organized and children’s self-evaluations tend to be more accurate compared to the ones provided by younger children (for a more in depth discussion, see Harter, 2012). In addition, although Tian et al. (2018)’s study showed the importance of POS for the psychological wellbeing of Chinese young adolescents, the extent to which children and adolescents can use the P Scale in a similar fashion as well as whether these two developmental groups differ in their mean-level of POS are still unclear.

In sum, the main goal of the present research was threefold. First, we aimed to analyze the psychometric properties of the P Scale between two groups of older children (years 5 and 6 of primary school) and early adolescents (years 7, 8, and 9 of secondary school) to ascertain the goodness of its hypothesized 1-factor structure. Second, we tested the measurement invariance of the P Scale across these two developmental groups to evaluate its developmental sensitivity to capture *true* (error-free) mean-level differences in POS across late childhood and early adolescence (see Malti et al., 2018b). Third, we examined the concurrent validity of the P Scale in relation to important developmental indicators of adjustment (i.e., prosocial behavior) and maladjustment (i.e., internalizing and externalizing problems) during late childhood and early adolescence using the Strengths and Difficulties Questionnaire (SDQ; Goodman, 1997). As a positive view of oneself and the surrounding world can offer the motivational and emotional resources to cope with the adversities of life, refrain individuals from harming others, and engage in (even costly) prosocial actions, we expected higher levels of POS to be associated with higher prosocial behavior (e.g., Luengo Kanacri et al., 2017) and lower levels of internalizing and externalizing problems (e.g., Caprara et al., 2018; Tian et al., 2018).

## Materials and Methods

### Participants

Data were collected from a total sample of 742 students (48% boys; age range from 9 to 15 years) from both primary (M*_age_* = 10.75, *SD* = 0.52, *n* = 421) and secondary schools (M*_age_* = 13.38 years, *SD* = 0.94, *n* = 321) in the United Kingdom. The participating schools were located in Liverpool.

### Procedure

Permission was obtained from the school principals and teachers. Only children who consented and obtained their parents’ informed written consent were included (98%). The self-report questionnaires of the P Scale and SDQ (in counterbalanced order) were distributed in classrooms in a paper-and-pencil format by trained researchers. Administration took approximately 25 min and children were debriefed at the end of the data collection. Ethical approval was obtained by the review board of Liverpool Hope University.

### Measures

#### POS

Participants rated the eight-item P Scale (Caprara et al., 2012; e.g., “I feel I have lots of things to be proud of,” “I look to the future with hope and optimism,” “I am satisfied with my life”), which measures individuals’ tendency to see their life and experiences with a positive orientation. Each item was rated using a 5-point scale from 1 (*strongly disagree*) to 5 (*strongly agree*) with higher scores reflecting greater positivity. Item 6 (“At times, the future seems unclear to me”) was reverse coded before running the statistical analyses. Omega (ω) reliability coefficients (McDonald, 1970) are reported in the results section.

#### SDQ

In order to assess the concurrent validity of the P Scale, students’ externalizing and internalizing problems, as well as their prosocial behavior were measured using the self-report version of the SDQ (Goodman, 1997). The SDQ is a widely used screening tool for children and adolescents to evaluate their developmental strengths and weaknesses. Although the SDQ was originally developed to assess five dimensions (i.e., prosocial behavior, emotional symptoms, peer problems, conduct problems, and hyperactivity/inattention), recent analyses of the SDQ indicated the benefit of considering two broader dimensions of externalizing (conduct problems and hyperactivity/inattention) and internalizing disorders (emotional symptoms and peer problems), in addition to prosocial behavior (see Caci et al., 2015). Omega reliability coefficients (McDonald, 1970) in the whole sample and in each age group (i.e., primary school students and secondary school students) were as follows: prosocial behavior (ωs = 0.760, 0.709, and 0.714, respectively); externalizing problems (ωs = 0.793, 0.784, and 0.802, respectively); internalizing problems (ωs = 0.774, 0.762, and 0.785, respectively).

## Results

### Factor Structure of the P Scale

We used confirmatory factor analysis (CFA) to ascertain the goodness of the hypothesized unidimensional factor structure of the P Scale. Maximum Likelihood with standard errors robust to non-normality (MLR) in M*plus* 8 (Muthén and Muthén, 1998–2017) was used to estimate the parameters and deal with missing data (6.1%)^1^. The MLR estimator requires the corrected χ^2^ difference test (Δχ^2^) for nested models (Muthén and Muthén, 1998–2017). To evaluate model fit, as the χ^2^ statistic is sensitive to sample size, we used the following rules-of-thumb: Comparative-Fit-Index (CFI) and Tucker-Lewis-Index values between 0.90 and 0.95 as indicators of moderate model fit, and CFI/TLI >0.95 as indicators of good model fit (Brown, 2015); Root-Mean-Square-Error-of-Approximation (RMSEA) with 90% Confidence Interval (CI), and Standardized-Root-Mean-Square-Residual (SRMR) values between 0.08 and 0.05 as indicators of moderate model fit and below <0.05 as indicator of good model fit (Iacobucci, 2010; Brown, 2015).

**FIGURE 1:**
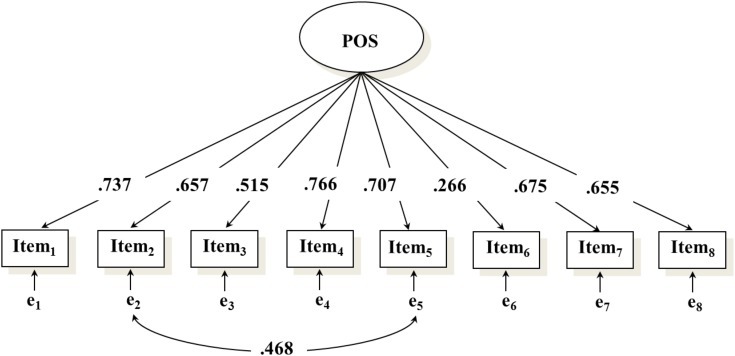
Confirmatory factor model of the P Scale. Standardized parameters are reported in the figure. Factor loadings and the correlation between the residual terms of item 2 and item 5 were statistically significant at *p* < 0.001. Item 1 (“I have great faith in the future”), item 2 (“I am satisfied with my life”), item 3 (“Others are generally here for me when I need them”), item 4 (“I look forward to the future with hope and enthusiasm”), item 5 (“On the whole, I am satisfied with myself”), item 6 reverse coded (“At times, the future seems unclear to me”), item 7 (“I feel I have many things to be proud of”), item 8 (“I generally feel confident in myself”).

The 1-factor solution had a poor fit to the data χ^2^ (20) = 174.439, *p* < 0.001, CFI = 0.901, TLI = 0.862, RMSEA = 0.103 [90% CI: 0.089, 0.117], SRMR = 0.041. A closer inspection of the modification indexes suggested the need (modification index = 106.147) to estimate the covariance among the residual terms of two items very similar in their wording (item 2 “I am satisfied with my life” and item 5 “On the whole, I am satisfied with myself”). The revised 1-factor model in which we estimated the correlation between the residuals of item 2 and item 5 (*r* = 0.468, *p* < 0.001) showed a better fit to the data *χ*^2^ (19) = 77.056, *p* < 0.001, CFI = 0.963, TLI = 0.945, RMSEA = 0.065 [90% CI: 0.050, 0.080], SRMR = 0.030. As shown in Figure 1, all the items had high standardized loadings except for the reverse-score item 6 (“At times, the future seems unclear to me”), which showed a small (i.e., below 0.40; Schaefer et al., 2015) standardized loading (λ = 0.266). The P Scale showed high reliability coefficients in the total sample (ω = 0.837), as well as in both primary school (ω = 0.806) and secondary school (ω = 0.835).

### Measurement Invariance Across Late Childhood and Early Adolescence

To test the invariance of the P Scale and its developmental sensitivity, we considered three nested models of measurement invariance (Vandenberg and Lance, 2000; Millsap, 2011) by imposing increasingly restrictive constraints on the factor loadings (λ) and intercept (τ) of the items of the P Scale. We first tested a configural invariance model in which both λs and τs were freely estimated across the two groups except for the marker item “I have great faith in the future” in which they were fixed to be λ = 1 and τ = 0 (Steenkamp and Baumgartner, 1998). Next, we tested metric (or weak) invariance in which the λs of the items were constrained to be equal across groups to ascertain whether the P Scale ranked the students based on their POS in the same way across late childhood (primary school) and early adolescence (secondary school). Lastly, we tested scalar (or strong) invariance by imposing both λs and τs to be equal across the two school levels to allow comparison at the level of latent means (Vandenberg and Lance, 2000). To test differences among configural, metric, and scalar invariance, we used the Δχ^2^ test as well as changes in CFI (ΔCFI) with a critical level of 0.01 (Cheung and Rensvold, 2002). As reported in Table 1, the P Scale reached strong scalar invariance (as attested by the non-significant change in the Δχ^2^ test), thereby allowing to meaningfully interpret as moderate-to-large (Cohen’s *d* = 0.776) the developmental difference at the latent (error-free) mean-level of positivity between primary (μ = 4.034) and secondary school students (μ = 3.536)^2^.

**Table 1:** Measurement invariance of the P Scale across late childhood and early adolescence (primary school vs. secondary school).

	χ^2^	*df*	*scf*	*p*	CFI	TLI	RMSEA (90%CI)	SRMR	MC	Δχ^2^	Δ*df*	*p*	ΔCFI
***P-Scale***													
1. Configural	106.769	38	1.193	<0.001	0.953	0.930	0.071 (0.055,0.087)	0.039					
2. Metric	110.160	45	1.233	<0.001	0.955	0.944	0.063 (0.048,0.078)	0.051	2 vs. 1	5.854	7	0.557	0.002
3. Scalar	123.682	52	1.203	<0.001	0.951	0.947	0.062 (0.048,0.076)	0.057	3 vs. 2	12.815	7	0.077	-0.004


*In addition to the *χ*^2^, the following fit indexes are reported: Comparative-fit-index (CFI); Tucker-Lewis-Index (TLI); Root-mean-square-error-of-approximation (RMSEA) with 90% confidence intervals (CI); Standardized-Root-Mean-Square-Residual (SRMR). *df* = degrees of freedom; *scf* = scaling correction factor; MC = model comparison.*

### Concurrent Validity of the P Scale Across Late Childhood and Early Adolescence

**FIGURE 2:**
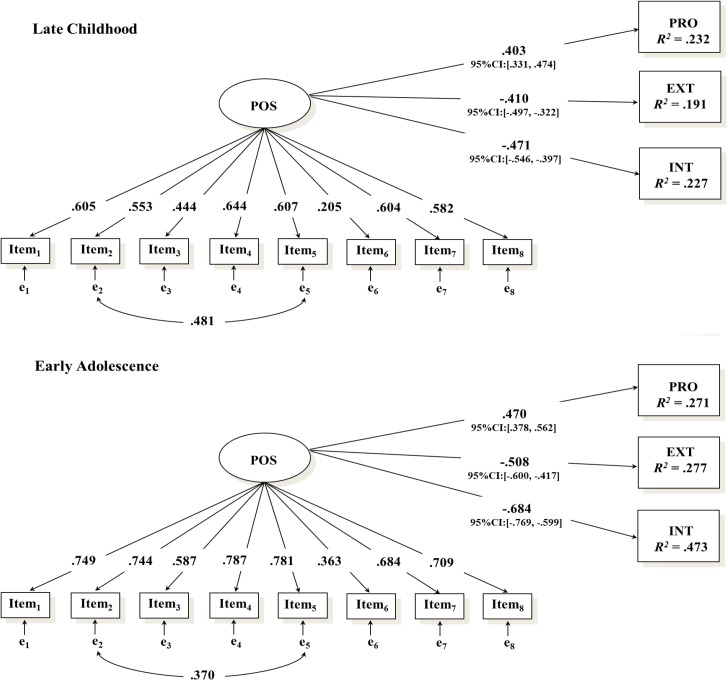
Concurrent validity of the P Scale. The effect of child’s gender on positivity (POS), prosocial behavior (PRO), externalizing (EXT), and internalizing problems (INT), as well as the correlations among PRO, EXT, and INT were estimated but not depicted for the sake of simplicity. The correlation coefficients among the residuals of the outcomes were as follows: PRO and EXT (*r*s = –0.100 and –0.082, *p* = 0.022 and 0.024, respectively, for late childhood and early adolescence), PRO and INT (*r*s = 0.182 and 0.199, both *p*s < 0.001, respectively, for late childhood and early adolescence), EXT and INT (*r*s = 0.274 and 0.037, *p* < 0.001 and *p* = 0.607, respectively, for late childhood and early adolescence). Standardized parameters are reported in the figure. Since unstandardized parameters were constrained to equality in the multiple-group approach, the size of the standardized parameters may differ across groups. Factor loadings, beta coefficients [with their 95% confidence intervals (CI)], and the correlation between the residual terms of item 2 and item 5 were statistically significant at *p* < 0.001.

We assessed the concurrent validity of the P Scale in each age group (primary school children vs. secondary school adolescents) by examining its relations with SDQ scores using a structural equation modeling (SEM) framework. In addition, we tested whether the structural paths were age-invariant by imposing equality constraints across the two groups. Gender (1 = boys; 2 = girls) was also included as a control variable. The constrained SEM, in which the structural parameters were fixed to equality, showed a moderate fit to the data χ^2^ (117) = 277.259, *p* < 0.001, CFI = 0.925, TLI = 0.916, RMSEA = 0.061 [90% CI: 0.052, 0.071], SRMR = 0.059, and was not statistically different Δχ^2^ (9) = 10.145, *p* = 0.339 from the unconstrained model in which the paths were freely estimated χ^2^ (108) = 267.480, *p* < 0.001, CFI = 0.926, TLI = 0.909, RMSEA = 0.064 [90% CI: 0.054, 0.073], SRMR = 0.053. As depicted in Figure 2, the results showed that, across both developmental groups, POS was positively associated with prosocial behavior and negatively associated with externalizing and internalizing problems, thereby attesting to the concurrent validity of P Scale across late childhood (primary school) and early adolescence (secondary school).

Children’s gender (1 = boys; 2 = girls) was not a significant predictor of POS (βs = -0.018 and -0.013, *p*s = 0.699, for late childhood and early adolescence, respectively). Finally, girls compared to boys scored higher on prosocial behavior (βs = 0.271 and 0.228, *p*s < 0.001, for late childhood and early adolescence, respectively) and internalizing problems (βs = 0.062 and 0.065, *p*s = 0.044 and 0.048, for late childhood and early adolescence, respectively), whereas they scored lower on externalizing problems (βs = -0.161 and -0.144, *p*s < 0.001, for late childhood and early adolescence, respectively).

## Discussion

Although previous studies pointed out to the relevance of POS for individuals’ psychological functioning from mid-adolescence to the elderly age, the extent to which the beneficial effect of POS could be extended to previous developmental stages has received little attention. Since POS has been found to be a key factor for people’s wellbeing, we aimed to investigate whether it could be reliably assessed in late childhood and early adolescence as well as its putative protective role during these two developmental phases. First, our results confirmed the plausibility of the hypothesized 1-factor structure of the P Scale (e.g., Caprara et al., 2012). However, one item (“At time, the future seems unclear to me”) reported a low standardized loading. Interestingly, this was the only reverse scored item. Since previous works indicated that children might have cognitive difficulties to respond appropriately to negatively worded items (Marsh, 1986), future use of the P Scale with children may consider positively rewording this item or delete it from the P Scale^3^. In addition, our analyses also indicated the need to estimate the covariance between the residual terms of two items (“I am satisfied with my life” and “On the whole, I am satisfied with myself”) to reach an acceptable model fit. Although the estimation of covariance among errors is not a recommended practice in factor analysis (Brown, 2015), the inclusion of this further parameter was likely due to a similar wording of the items (i.e., method effects) rather than to the presence of additional substantive latent factors (for similar results on the factor structure of the P Scale see: Caprara et al., 2012; Tian et al., 2018). Taken together, however, these findings may suggest the importance of considering rewording some items to reduce unnecessary, additional shared variance among the indicators composing the P Scale. For example, item 5 could be reworded as “I am happy for who I am” to avoid the double use of the wording “I am satisfied with...” in the same scale. Furthermore, considering the ability of children to reflect on their future using the annual cycle as representation of the time (Friedman, 2000), Item 4 (“I look forward to the future with hope and enthusiasm”) could be reworded to capture a more precise time lag (e.g., “next year”).

Our results also confirmed the strong measurement invariance of the P scale for the two different age groups (i.e., late childhood and early adolescents), thereby attesting to the developmental sensitivity of the P Scale to capture true, latent mean-level differences in POS. In detail, we found that children reported higher mean levels of POS compared to early adolescents. Although this difference could be attributed to a children’s *positive bias* to see their life in an overly positive way (likely due to their cognitive limitations to properly compare between real and ideal situations; Harter, 2012), it may also indirectly reveal information about the drop in psychological wellbeing during the transition from childhood to adolescence. A consistent number of studies, indeed, clearly showed that children tend to experience higher wellbeing than adolescents in both general (Ronen et al., 2016) and domain specific evaluations (e.g., at school; López-Pérez and Fernández-Castilla, 2018). Some authors (e.g., Denscombe, 2000) have explained such differences considering the challenges that adolescents experience, especially in the school context (e.g., higher academic demands), which may threaten their sense of autonomy, competence, and relatedness, thereby ultimately influencing their POS and wellbeing. Future longitudinal studies are needed to better understand why both POS and wellbeing decrease with age and to what extent their declining trajectories influence each other.

Finally, we also found evidence for the concurrent validity of the P Scale in the two developmental phases considered. In line with previous studies attesting to the protective role of POS during adolescence (e.g., Luengo Kanacri et al., 2017) and adulthood (e.g., Alessandri et al., 2014), higher POS was consistently linked to higher prosocial behavior and lower internalizing and externalizing problems across late childhood and early adolescence.

### Limitations and Future Research

Despite a number of strengths, our study was not without limitations. First, we recognize that our study was cross-sectional, thereby limiting the conclusions that we can draw in terms of developmental differences. Hence, future studies should use a longitudinal approach to have a more valid assessment of possible developmental shifts in POS during the transition from late childhood to early adolescence. Second, the measures used in the study were all self reports. Although self reports are the optimal source of information for POS (Caprara et al., 2018), future studies should adopt a multi-informant approach (e.g., teachers, peers, etc.,) for prosocial behavior and externalizing/internalizing problems to mitigate the possibility of inflated effects due to the same-informant bias. Third, in the present study, we only evaluated the psychometric properties of the P Scale and the role of POS with children who were 9-year-old or older. Future studies should consider how POS could be evaluated in a developmentally appropriate way with younger children to expand our knowledge on how POS develops from an early age. Moreover, although POS has been found to be a protective factor across different developmental phases, it has not been evaluated whether extreme values of POS could have a detrimental effect as it may lead to a distorted perception of the self and one’s own reality. Since emotion research has found that an excessive preference for positive emotions is linked to lower well-being (Ford and Tamir, 2012) and clinical symptoms (Ford et al., 2015), we urge future research to clarify the possible “side” effect of having an excessive POS. Finally, we also recognize that we did not evaluate the divergent validity of the P Scale in relation to other key variables such as personality traits, positive affect, and self-esteem. Although previous research (mostly with adults and late adolescents) has already shown the unique contribution of POS, more work is needed to clarify its divergent and predictive validity during early developmental windows (e.g., childhood and early adolescence) as well as across clinical versus non-clinical samples.

## Conclusion

As the reliable assessment of individual strengths and their timely promotion among children is at the core of many SEL intervention programs (Malti et al., 2018a), the present study provided evidence about the protective role of POS and the goodness of the P Scale during late childhood and early adolescence. Although more research is essential to further ascertain the validity (e.g., divergent and predictive) of the P Scale, current findings suggest that the P Scale is a promising, short and easy-to-use self-report instrument to be included in the “tool-box” of psychologists and practitioners working in the field of prevention science and/or promotion of wellbeing.

## Ethics Statement

This study was carried out in accordance with the recommendations of the American Psychological Association (APA) guidelines with written informed consent from all subjects. All subjects gave written informed consent in accordance with the Declaration of Helsinki. The protocol was approved by the review board of Liverpool Hope University.

## Author Contributions

AZ conceived of the study, participated in its design, drafted the manuscript, performed the statistical analysis, and interpretation of the data. BL-P conceived of the study, participated in its design, drafted the manuscript, and interpretation of the data. FC and JK drafted the manuscript and performed the statistical analysis. GC helped to draft the manuscript. All authors read and approved the final manuscript.

## Conflict of Interest Statement

The authors declare that the research was conducted in the absence of any commercial or financial relationships that could be construed as a potential conflict of interest.

## Footnotes

1 We used the MLR estimator as the Mardia’s test for multivariate normality was statistically significant (*p* < 0.001) for both skewness and kurtosis. In addition, item 3, “Others are generally here for me when I need them,” showed skewness (1.014) and kurtosis (1.150) higher than |1|.

2 We also tested a model in which the latent means were constrained to be equal across groups. As the constrained model *χ*^2^ (53) = 192.509, *p* < 0.001, CFI = 0.904, TLI = 0.899, RMSEA = 0.085 [90% CI: 0.072, 0.098], SRMR = 0.100 was statistically different from the unconstrained scalar model reported in Table 1 [Δχ^2^ (1) = 107.873, *p* < 0.001], we concluded that the two groups were characterized by true developmental differences in terms of their POS.

3 We also tested our models by removing item 6 from the analyses and the results were virtually identical.
